# Trends in 3D Printing Processes for Biomedical Field: Opportunities and Challenges: An Editorial Retrospective

**DOI:** 10.3390/biomedicines12112612

**Published:** 2024-11-15

**Authors:** M. R. Mozafari

**Affiliations:** Australasian Nanoscience and Nanotechnology Initiative (ANNI), 8031 Monash University LPO, Clayton, VIC 3168, Australia; dr.m.r.mozafari@gmail.com

On 8 August 1984, Chuck Hull (Charles W. Hull) filed US patent number US4575330A with the title “Apparatus for production of three-dimensional objects by stereolithography” [1]. Hull’s apparatus was in fact the world’s first 3-dimensional printer device. This invention opened the way to a novel method of industrial fabrication called ‘additive fabrication’, as opposed to the formerly utilized ‘subtractive fabrication’. Additive fabrication is basically a mechanical process whereby solid objects are created by ‘printing’ successive layers of material to replicate a computer-generated model. Accordingly, 3D objects are created by adding layers of material starting from nothing instead of cutting, carving, or drilling extra sections from a full block until the desired shape or object is obtained. This method of fabrication is known as 3D printing with reference to a similar technology used in ink-jet printers [2].

Also known as rapid prototyping, 3D printing technology possesses a significant impact on all aspects of our lives and is playing an increasing important role in many disciplines, including art, engineering, manufacturing, medicine, and education [3]. Along with the continual advancements in science and technology, the scope of 3D printing is undergoing constant expansion, penetrating an array of different pharmaceutical, biomedical, and food sectors in particular. Applications of this technology in life science and medicine have gradually become a multi-disciplinary approach called 3D bioprinting that is being employed in biomimetic scaffolds, various human implants, drug testing models, and controlled drug release strategies [4,5]. It is undeniable that this contemporary technology plays an increasingly vital role in the modern biomedical applications based on which it is selected as the topic of this Special Issue published in the Biomedicines journal.

Several manuscripts were submitted for consideration to the Special Issue, and all of them were subject to a rigorous review process. In total, seven original research papers were finally accepted for publication and inclusion in this contemporary Special Issue as listed.

As shown in Table 1, the contributions covered different aspects of 3D bioprinting, including 3-dimensional printing of heart components, brain models, bone scaffolds, and pharmaceutical tablets with improved properties. An interesting aspect of the published papers in this Special Issue is that each of them was authored by scientists and researchers from different departments and research institutes.

It is worth mentioning that while the first two contributions evaluated their formulated 3D structures using in vitro cell/tissue models [6,7], the third contribution evaluated the osteogenic and angiogenic properties of 3D-printed isosorbide-based CSMA-2 gyroid scaffolds manufactured via the digital light processing method in vivo. Profound development has been achieved in understanding the relationships between the 3D-printing processes and the structures, properties, and applications of the created or developed objects. Continuing improvements of novel biomaterial inks have enabled the production of models and in vitro implants capable of achieving certain levels of success in the in vivo (using animal models) and preclinical trials. Significant progress in cell biology and computational design has aided in achieving the latest milestones with planned tissue- or organ-mimetic constructs, which possess certain levels of functionality. Nevertheless, bio-fabricated structures and models still have a long way to go until attaining the clinical phases of development [8,9,10,11].

I hope that the readers of this Special Issue find the broad and innovative contents of the manuscripts useful in their research and studies. Considering the quality and novelty of the articles published in this Special Issue, I genuinely hope that these papers will inspire scientists working in the fascinating field of 3D bioprinting.

## Figures and Tables

**Table 1:** Analysis of the published contributions in the Special Issue. Note the multidisciplinary nature of the listed research.

Contribution No.	Focus/Aim	Primary Research Discipline	Secondary Research Discipline
1	3D models for cardiac ablation	Cardiac Surgery	Heart Electrophysiology
2	Bone reconstruction (based on β-TCP matrices produced using top-down DLP * 3D printer)	Oral and Maxillofacial Surgery	Ceramic Materials
3	New bone formation and neovascularization of 3D-printed structures evaluated in vivo	Biomaterials and Tissue Engineering	Tissue Regeneration Engineering
4	Bone tissue engineering (based on a combined computational and experimental study)	Biomedical Engineering	Computation-Based Science and Technology
5	Formulation of filament-based 3D printlets to enhance the therapeutic effects of apigenin	Health Sciences	Pharmaceutics
6	Formulation of 3D-printed tablets with enhanced properties	Pharmaceutics	Chemistry
7	Development of a real-life brain model using 3D printing technology	Neurosurgery	Orthopedics

* DLP: digital light processing.
